# Age-related disparities in national maternal mortality trends: A population-based study

**DOI:** 10.1371/journal.pone.0316578

**Published:** 2025-01-03

**Authors:** Ryan S. Huang, Andrea R. Spence, Haim A. Abenhaim

**Affiliations:** 1 Temerty Faculty of Medicine, University of Toronto, Toronto, Ontario, Canada; 2 Center for Clinical Epidemiology, Lady Davis Institute, McGill University, Montreal, Quebec, Canada; 3 Department of Obstetrics & Gynecology, Jewish General Hospital, McGill University, Montreal, Quebec, Canada; University of Tennessee Knoxville, UNITED STATES OF AMERICA

## Abstract

**Objective:**

An upward trend in maternal age has been observed in the United States (US) over the last twenty years. The study objective was to examine the association of maternal age with maternal mortality in the US and examine temporal trends in mortality by maternal age.

**Methods:**

A retrospective population-based analysis in the US between 2000–2019 was conducted using records from the Centers for Disease Control and Prevention’s “Mortality Multiple Cause” and “Birth Data” files. Annual incidence and period trends in maternal deaths were calculated using the annual maternal deaths over annual live births across age groups. Multivariate logistic regression models were used to estimate the association between maternal age and risk of maternal mortality and calculate temporal changes in risk of mortality over the study period.

**Results:**

Between 2000–2019, 21,241 deaths were observed in women during pregnancy and childbirth for an average incidence of 26.3 maternal deaths/100,000 births (95% CI 21.8–31.2). Of all deaths, 6,870 (32.3%) were in women ≥35 years, while only 15.1% of live births were attributed to women ≥35 years. Compared with women 25–29 years of age, there was a significantly greater risk of maternal mortality among women 35–39 (OR 1.60, 1.53–1.67), 40–44 (3.78, 3.60–3.99), 45–49 (28.49, 26.49–30.65) and 50–54 (343.50, 319.44–369.37). Risk of mortality increased over time, with the greatest rise in women ≥35 years.

**Conclusion:**

In the US, maternal mortality increased during the past two decades, especially in women ≥35 years. Given these findings, targeted strategies to reduce the increasing maternal mortality should become a priority.

## Introduction

Maternal mortality remains high, with estimates by the World Health Organization (WHO) that over 300,000 women die each year from pregnancy or childbirth-related complications [1]. While maternal mortality has decreased in most developed countries over the past two decades, recent reports show maternal mortality has increased in the United States (US) [2, 3]. Maternal mortality is a critical measure of a country’s healthcare efficacy and carries profound impacts on the families affected and society at large [4].

In several high-income countries, there has been a notable decrease in the number of births among adolescents aged 10 to 19 years. Conversely, a notable shift towards higher maternal ages is emerging [5]. This demographic change has sparked growing concerns over the potential negative outcomes for mothers aged 35 and above. These concerns are rooted in the established link between advanced maternal age and an escalation in complications during childbirth, such as preterm deliveries and stillbirths [6–8]. Despite this, there remains a scarcity of comprehensive data on the relationship between advancing maternal age and maternal mortality rates [9].

Moreover, existing research on maternal mortality has predominantly concentrated on deaths due to obstetric complications, neglecting those resulting from accidental and incidental causes [10]. This could stem from the WHO’s maternal death definition, which exclusively accounts for obstetric-related causes [11]. Nevertheless, substance abuse, homicides, as well as other non-obstetric factors contribute significantly to the number of women dying during pregnancy and childbirth [12–14]. Therefore, to provide a complete overview of maternal mortality across different age demographics and to guide public health measures more effectively, it is crucial to consider all contributing causes of death.

The objective of our study was to carry out a nationwide analysis to examine the association of maternal age with maternal mortality in the US and examine temporal trends in mortality by maternal age.

## Materials and methods

### Study design

We conducted a 20-year retrospective population-based study using birth and mortality data from the National Center for Health Statistics (NCHS) and the National Vital Statistics System (NVSS) under the auspices of the Center for Disease Control and Prevention (CDC). In the US, birth certificates are obligatory for all births and are merged by the NCHS to create the “Birth Data” files. Mortality records, on the other hand, originate from official death certificates completed by medical professionals, which detail the primary and any secondary causes of death [15]. These certificates are initially collected at vital statistics offices within each state before being consolidated in the NVSS, resulting in the formation of the “Mortality Multiple Cause” data files. The NVSS thus ensures the inclusion of death data from every state, authenticated by medical professionals.

In this study, maternal death was defined as any woman’s death occurring during pregnancy or childbirth within 42 days of pregnancy termination from any cause, including those where pregnancy exacerbates pre-existing conditions or those resulting from accidental or unrelated events. This definition broadens the WHO definition by including accidental and incidental deaths, thereby accounting for all maternal deaths associated with pregnancy or childbirth [11].

### Cohort creation

To create the study cohort, we collated all live birth occurrences in the US from the period of January 2000 to December 2019, using data from the “Birth Data” files. Subsequently, we employed the WHO classification of International Classification of Diseases (ICD)-10 codes codes [16], to identify deaths during pregnancy and childbirth within 42 days post-pregnancy termination. In particular, the following codes were used: A34, O00-O95, and O98-O99. The code A34 pertains to obstetrical tetanus, a rare but serious condition that can occur during pregnancy. The series of codes from O00-O95 denote various obstetric deaths, including those due to complications predominantly related to the puerperium, and those specified as relating to pregnancy, labor, and delivery. The codes O98-O99 cover other diseases classified elsewhere but complicating pregnancy, labor, and puerperium. Our inclusion criteria mandated the presence of the ICD-10 codes as either a primary or secondary contributing cause of death listed in the “Record-Axis” section of the dataset, which denotes all primary and secondary causes of each death. The ensuing dataset amalgamated the derived maternal mortality data from the NVSS and live birth data from the NCHS. All data was accessed on May 15^th^, 2022, and no identifying data was accessible.

### Statistical analyses

To investigate the associations in our study, we examined several key variables. Maternal age was recorded as the mother’s age at the time of delivery. For categorizing maternal age, we adopted a standard demographic approach to group ages into defined categories that allow for comparison across different age brackets. Specifically, maternal age was classified into the following categories: under 15, 15–19, 20–24, 25–29, 30–34, 35–39, 40–44, 45–49, and 50–54 years. Each category encompasses a span of five years, providing a clear and systematic means of analyzing age-related trends and outcomes. Age was used as a categorical variable in the analyses. The main outcome variable of our study was maternal death, which was defined as any death of a woman while pregnant or within 42 days of the end of pregnancy.

A three-phase approach was implemented for analysis. Initially, we calculated both the overall and annual rates of maternal mortality per 100,000 live births for the years 2000 through 2019. Subsequently, mortality rates specific to each age category were computed and evaluated against the distribution of live births. The Shapiro-Wilk test was used to assess the normality of data. To address the distribution’s skewness due to increased mortality risks in older age brackets, maternal mortality rates/100,000 births were plotted on a logarithmic scale with a base of ten. The next phase involved the application of logistic regression models to assess the association between maternal age and mortality risk through computing odd ratios (OR) and 95% confidence intervals (CIs). The models were adjusted for the potential confounding effects of calendar year and maternal race, categorized according to the classifications used in the data source [17]. Finally, we estimated the annual change in maternal mortality rates through the slope of maternal mortality per 100,000 live births over year. Race-adjusted logistic regression models that treated year of maternal death as a continuous independent variable, were also used to examine the time-based shifts in the incidence of maternal mortality across different age groups. All analyses were conducted using SAS 9.4 statistical software. P-values <0.05 were considered statistically significant.

### Ethics approval

Based on the Tri-Council Policy of 2018, institutional ethics approval was not required for this study as it was only based on data from a publicly available database [18].

## Results

**Fig 1:**
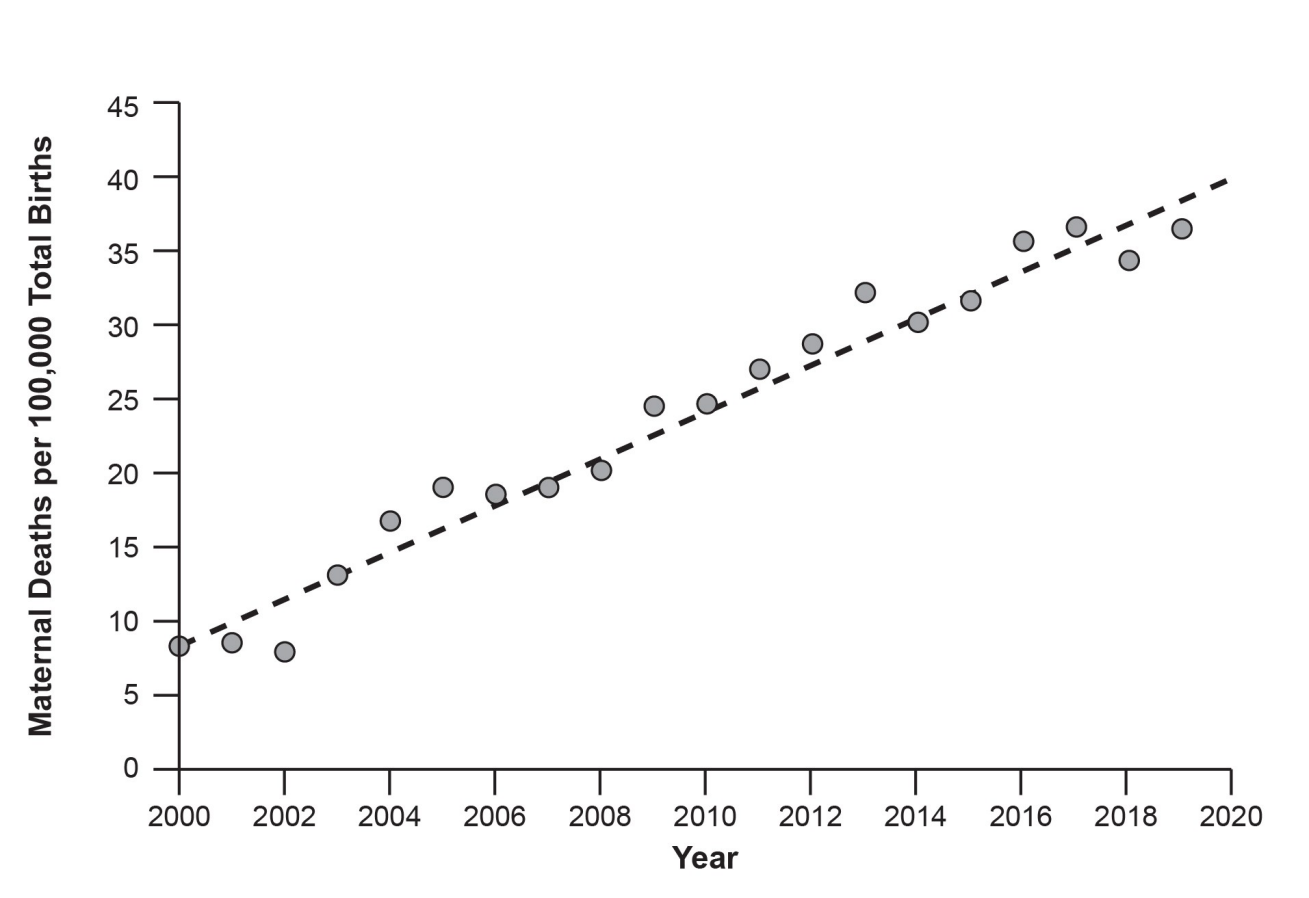
Annual Incidence of Maternal Deaths per 100,000 Deliveries from 2000–2019 in the US.

Between 2000 and 2019, 21,241 maternal deaths and 80,710,348 live births occurred in the US. Most deaths arose from obstetric complications (n = 13,907, 65.5%), while 34.5% of deaths occurred from non-obstetrical causes (n = 7,334). As shown in Fig 1, an upward trend in maternal deaths per 100,000 deliveries was observed over the study period.

**Fig 2:**
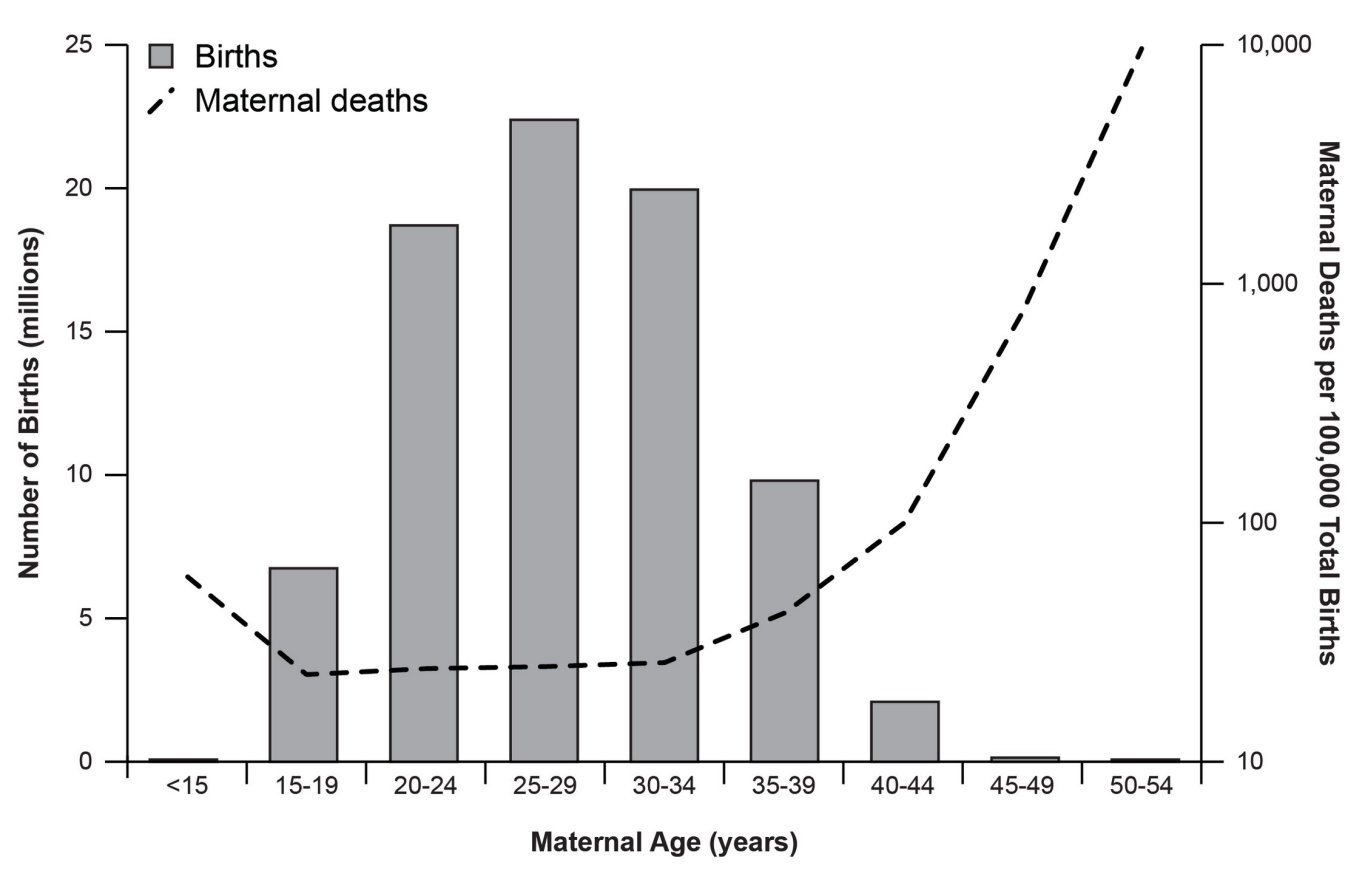
Distribution of maternal age and mortality in the US from 2000–2019. Maternal Deaths/100,000 Births were plotted on a log scale with a base of 10.

Fig 2 presents the distribution of maternal age and the corresponding rates of maternal deaths per 100,000 births within those age brackets. The greatest proportion of births occurred in mothers aged 25–29 years (n = 22,582,967, 28.0%) and the number of births decreased with increased age. The number of maternal deaths per 100,000 births remained relatively stable for maternal ages spanning 15–34; however, there was a marked exponential increase in the mortality rate with increased age, peaking among those aged 50–54.

Table 1 illustrates the breakdown of maternal deaths, the aggregate of live births and the maternal mortality ratios segmented by age categories, alongside the association between maternal age and the occurrence of maternal deaths. Although only 15.1% (n = 12,218,318) of all live births were attributed to a maternal age of 35 years or older, 32.3% (n = 6,870) of all maternal deaths occurred in women ≥35 years. Compared to women aged 25–29, women aged 35 years or older were found to be at a significantly greater risk of maternal mortality with the risk increasing in each older age group and reaching the highest risk in women aged 50–54. Similarly, the risk of maternal mortality was found to be 3-times greater in women under 15 compared to women aged 25–29, but these deaths only made up 0.2% (n = 52) of all maternal deaths.

**Table 1:** Maternal deaths and live births in the US from 2000–2019.

Cohort	Maternal Deaths N (%)	Live Births from the General US Population N (%)	Maternal Deaths per 100,000 Births (95% CI)	Adjusted Odds Ratio* (95% CI)	P-value
**All**	21,241	80,710,348	26.3 (21.8–31.2)		
**Age**					
< 15	52 (0.2)	95,470 (0.1)	54.5 (44.9–97.9)	3.12 (2.38–4.10)	<0.0001
15–19	1,409 (6.6)	6,830,109 (8.5)	20.6 (18.5–28.7)	1.10 (1.04–1.17)	<0.0001
20–24	3,908 (18.4)	18,840,439 (23.3)	20.7 (17.5–25.9)	1.04 (1.01–1.09)	<0.0001
25–29	4,673 (22.0)	22,582,967 (28.0)	20.7 (17.0–24.3)	Reference	
30–34	4,215 (19.8)	20,143,045 (25.0)	20.9 (17.6–23.6)	1.05 (1.03–1.10)	<0.0001
35–39	3,361 (15.8)	9,930,420 (12.3)	33.8 (28.8–37.8)	1.60 (1.53–1.67)	<0.0001
40–44	1,705 (8.0)	2,137,394 (2.6)	79.8 (65.1–91.3)	3.78 (3.60–3.99)	<0.0001
45–49	863 (4.1)	138,733 (0.2)	622.1 (413.8–755.6)	28.49 (26.49–30.65)	<0.0001
50–54	941 (4.4)	11,771 (0.0)	7,994.2 (4,410.4–10,036.5)	343.50 (319.44–369.37)	<0.0001

*Regression model adjusted for year and race

Table 2 presents the progression in maternal mortality rate by age from the period of 2000 to 2019. Throughout the two decades, the greatest annual increase per 100,000 births in maternal mortality was observed in women aged 50–54.

**Table 2:** Annual change in the maternal death rate from 2000–2019 in the US population, stratified by age.

Cohort	Annual Increase per 100,000 Births	Adjusted Odds Ratio* (95% CI)	P-value
**All**	1.6	1.07 (1.06–1.07)	<0.0001
**Age**			
< 15	6.3	1.11 (1.06–1.17)	<0.0001
15–19	1.8	1.07 (1.07–1.08)	<0.0001
20–24	1.5	1.05 (1.05–1.06)	<0.0001
25–29	1.3	1.05 (1.05–1.06)	<0.0001
30–34	1.1	1.05 (1.04–1.06)	<0.0001
35–39	1.5	1.05 (1.04–1.05)	<0.0001
40–44	4.1	1.09 (1.08–1.10)	<0.0001
45–49	34.9	1.33 (1.31–1.36)	<0.0001
50–54	486.0	1.56 (1.32–1.80)	<0.0001

*Regression model adjusted for race

Table 3 contrasts the top ten reported underlying causes of maternal death between 2000 and 2019. In 2000, all underlying causes of death were obstetric in nature. Conversely, in 2019, the frequency of reporting non-obstetric causes, including accidental poisoning and assault, had increased.

**Table 3:** Underlying causes of maternal death in 2000 compared to 2019.

Cause of Death	N (%)
**2000**	
O88.1 (Amniotic fluid embolism)	47 (11.2)
O90.3 (Cardiomyopathy in the puerperium)	27 (6.4)
O99.8 (Other specified diseases and conditions complicating pregnancy, childbirth, and the puerperium)	26 (6.2)
O14.9 (Pre-eclampsia, unspecified)	23 (5.5)
O99.4 (Diseases of the circulatory system complicating pregnancy, childbirth, and the puerperium)	18 (4.3)
O14.1 (Severe pre-eclampsia)	16 (3.8)
O00.9 (Ectopic pregnancy, unspecified)	15 (3.6)
O75.9 (Complication of labour and delivery, unspecified)	14 (3.3)
O16 (Unspecified maternal hypertension)	12 (2.9)
O15.9 (Eclampsia, unspecified as to time period)	11 (2.6)
Total	420
**2019**	
O26.8 (Other specified pregnancy-related conditions)	174 (9.6)
X42 (Accidental poisoning by and exposure to narcotics and psychodysleptics [hallucinogens], not elsewhere classified)	147 (8.1)
X44 (Accidental poisoning by and exposure to other and unspecified drugs, medicaments, and biological substances)	111 (6.1)
O99.8 (Other specified diseases and conditions complicating pregnancy, childbirth, and the puerperium)	96 (5.3)
X95 (Assault by other and unspecified firearm discharge)	86 (4.7)
O99.4 (Diseases of the circulatory system complicating pregnancy, childbirth, and the puerperium)	72 (4.0)
V89.2 (Person injured in unspecified motor-vehicle accident, traffic)	57 (3.1)
X70 (Intentional self-harm by hanging, strangulation and suffocation)	41 (2.3)
O90.3 (Cardiomyopathy in the puerperium)	32 (1.8)
O95 (Obstetric death of unspecified cause)	30 (1.6)
Total	1820

## Discussion

Our study sought to report population-based maternal mortality incidence and trends in the United States. From 2000–2019, the incidence of maternal mortality rose, and this trend coincided with increases in the proportion of pregnancies to older mothers. Increasing maternal age was found to be associated with a higher risk of maternal mortality with the greatest risk in the oldest age group of 50–54, while mothers under the age of 15 were also found to be at a greater risk.

Similar to our study, other recent population-based studies have also demonstrated a rise in maternal mortality in the US [19–21]. The maternal mortality ratio was reported to have increased from 7.2 deaths per 100,000 births in 1987 to 17.8 deaths per 100,000 births in 2009 [22]. However, only deaths from obstetrical complications were considered in these studies leading to a significant underreporting of total deaths in pregnant women. Our study found 26.1 deaths per 100,000 births in 2009, as opposed to 17.8 deaths per 100,000 births, when including non-obstetrical causes of death and draws attention to the importance of considering accidental and incidental causes of death. From 2000–2019, we found the number of deaths during pregnancy from both obstetrical and non-obstetrical causes to have increased from 10.9 to 39.3 deaths/100,000 births. This rise signals potential gaps in the healthcare system, disparities in access to essential maternal health services, and variations in the quality of care. The increase in maternal mortality rates could be linked to systemic issues such as the fragmented delivery of healthcare, socioeconomic barriers limiting access to prenatal care, and challenges in managing preventable and treatable conditions like preeclampsia and hemorrhage [23, 24]. Moreover, the wider adoption of computerized data [25], changes in the coding for causes-of-death post-ICD-10 implementation [26], and the introduction of a pregnancy checkbox on US death certificates [27], have likely led to more accurate reporting of maternal mortality. Concurrently, our analysis demonstrated that accidental and incidental causes of maternal death, such as those resulting from substance abuse, motor vehicle accidents, homicide, and other non-obstetric factors were reported more frequently in the year 2019 compared to 2000. This aligns with recent reports showing an increased trend in non-obstetric maternal mortality over the past two decades, particularly in high-income countries where advances in obstetric care have reduced direct obstetric deaths [28]. By considering these broader causes of death, we emphasize the necessity of a holistic approach to maternal health that encompasses management of pregnancy and delivery, as well as societal measures to prevent accidents, and healthcare policies aimed at improving access and quality of care for all pregnant women [29, 30].

Our study observed a greater risk of maternal mortality in women younger than 15, and in women 35 years or older, as well as an increase in maternal deaths in these groups over the last 2 decades. These results are similar to a study by Callaghan and Berg, who found a higher pregnancy-related mortality ratio for older women aged 40 years or older regardless of parity, the extent of prenatal care, and level of education [31]. The trend of older maternal age in pregnancy observed in the US is likely to have direct effects on the increase in maternal mortality [32]. Older women are becoming increasingly represented in the pregnant population with a 74% increase in pregnancy rates in women aged 35–39 years and a 38% increase in women aged 40 years and older from 1976–1997 in the US [33]. Studies have shown older women are more likely to have chronic morbidities prior to conception, such as hypertension, diabetes, and various autoimmune diseases. These pre-existing conditions also render them at greater likelihood of developing peripartum adverse conditions [34, 35]. For instance, cardiomyopathy, thromboembolic disease, and postpartum hemorrhage are especially prevalent in older women [36, 37]. Moreover, pregnant women in their mid-30s and older have a higher risk of experiencing obstetric acute renal failure [38], providing evidence that baseline variables associated with aging such as lower cardiac output and hypertension, could make older mothers less able to adapt to the normal physiological changes occurring during pregnancy and therefore, increasing their risk of death. Increasing oxidative stress with aging has also been shown to be a key factor of placental insufficiency and maternal mortality [39]. In addition to biological risks associated with advanced maternal age, these age groups must navigate complex social determinants of health that can impact their maternity experience. Socioeconomic status, educational opportunities, and healthcare accessibility play crucial roles in maternal outcomes. These factors, combined with the pressures of balancing career and family life, can delay childbearing and lead to gaps in healthcare coverage or continuity of care, especially for women transitioning from private to public insurance or those without insurance [40]. Furthermore, disparities in healthcare access, such as the availability of specialized maternal care and proximity to healthcare facilities, can exacerbate the risk of peripartum complications [41].

Over the past 2 decades, the growth in availability of assisted reproductive technology (ART) has allowed more women 35 years or older, when fertility is on the decline, to become pregnant [42]. In the US, ART has been associated with an elevated risk of maternal mortality [43, 44], and hence, its use may have contributed to the elevated maternal mortality observed in our study among older women. However, the database used for this study did not include data regarding mode of conception. Recognition of the heightened risk of death among older pregnant women is needed to properly counsel women planning to become pregnant after their mid 30’s and to better inform their care before, during and after pregnancy.

Previous studies have highlighted the association between adolescent pregnancy and an elevated risk of adverse perinatal outcomes, such as preterm delivery, stillbirth, and neonatal death [45–47]. The occurrence of these adverse outcomes may contribute to the increased maternal mortality observed, as complications arising from preterm delivery or stillbirth can lead to critical maternal health challenges. In the US, these issues are compounded by the higher and increasing rates of adolescent births compared to other developed countries [48]. Current evidence highlights a large role of social determinants of health such as socioeconomic status on the high rate of adolescent births in the US [49]. These findings should divert focus to efforts that address underlying socioeconomic factors to reduce adolescent pregnancy, which not only increases the risk of maternal mortality, but is central to the promotion of women’s educational, social, and economic development. Increasing the availability of contraceptives and increasing accessibility to therapeutic abortions should also further decrease the number of adolescent pregnancies [50].

While our study is the largest nationwide analysis of maternal outcomes in relation to maternal age in the US, it is not without its limitations. The “Mortality Multiple Cause” data files were constrained by a limited set of demographic variables and lacked others that might impact maternal mortality such as body mass index and assisted reproductive technology (ART). Despite these gaps, race was available and accounted for in our regression models to mitigate potential biases. Moreover, the reliance on vital statistics data and shifts in data collection methods over time, such as the introduction of the pregnancy checkbox on death certificates in 2003, could have led to misclassification of pregnancy-associated deaths. This issue is particularly relevant for deaths ascribed to broad categories like “Other pregnancy-related condition,” which could be refined in the future by mandating the inclusion of more precise ICD-10 codes. Additionally, we reported maternal deaths per live births rather than per total pregnancies. Future research should examine pregnancy-related mortality ratios that include total pregnancies in the denominator to provide a more holistic view of maternal mortality. The robustness of our study is supported by its considerable sample size, which comprehensively encompasses all 80 million live births in the US over two decades and all maternal deaths authenticated by medical professionals. This allowed for the detection of significant associations between maternal age and mortality. These strengths not only reinforce the credibility of our results, but also underscore the potential for these findings to be applicable to the broader American populace.

## Conclusions

In conclusion, maternal mortality rose in the US over the past two decades, with the highest risk observed in adolescents younger than 15 years and women 35 years or older. These results highlight the need for medical providers to recognize the risk of death borne by women who become pregnant at either extreme of the reproductive age span in order to provide appropriate care. Further, the increased use of ART in older women, as well as underlying socioeconomic factors to reduce adolescent pregnancy need to be addressed. Our results highlight rising maternal mortality in the United States as becoming an increasingly important concern that should be addressed as a public health priority.
